# Association of lipid-lowering drugs with osteoarthritis outcomes from a drug-target Mendelian randomization study

**DOI:** 10.1371/journal.pone.0293960

**Published:** 2024-02-28

**Authors:** Weiwei Ma, Honggu Chen, Zhiwen Zhang, Yong Xiong

**Affiliations:** 1 Affiliated Hospital of Hubei University of Chinese Medicine, Wuhan, China; 2 Hubei Provincial Hospital of Traditional Chinese Medicine, Wuhan, China; 3 Hubei Provincial Institute of Traditional Chinese Medicine Wuhan, China; 4 The Affiliated Hospital of Jiangsu University, Zhenjiang, Jiangsu, China; 5 School of Acupuncture-Moxibustion and Orthopedics, Hubei University of Chinese Medicine, Wuhan, China; Catholic University of Brasilia, BRAZIL

## Abstract

**Background:**

Osteoarthritis (OA), a prevalent musculoskeletal disorder, has been suggested to have a potential association with metabolic syndrome, particularly lipid metabolism. Studies exploring the effects of lipid-lowering drugs on OA have yielded conflicting results.

**Objective:**

This study employed a drug-targeted Mendelian randomization approach to investigate the association between genetically predicted lipid-modulating effects of commonly targeted lipid-lowering agents and the risk of OA, with the aim of providing a theoretical foundation for the use of lipid-lowering drugs in OA treatment.

**Methods:**

Employing Mendelian randomization (MR) analysis, we examined the potential causal relationship between lipid-lowering drugs and OA. Genetic variants associated with LDL cholesterol levels were selected from the GWAS summary data, and a series of statistical analyses, including inverse-variance weighted (IVW), weighted median (WM), and MR-Egger, were performed to estimate causal effects.

**Results:**

We observed significant associations between genetically proxied lipid-lowering drug targets and OA risk. Notably, HMGCR-mediated LDL cholesterol showed an association with overall OA of the hip or knee (OR = 0.865, 95%CI: 0.762 to 0.983, *p* = 0.026, q = 0.07) and knee osteoarthritis specifically (OR = 0.746, 95%CI: 0.639 to 0.871, *p* = 2.180×10^−4^, q = 0.004). PCSK9-mediated LDL cholesterol also demonstrated an association with OA of the hip or knee (OR = 0.915, 95%CI: 0.847 to 0.988, *p* = 0.023, q = 0.07) and knee osteoarthritis (OR = 0.901, 95%CI: 0.821 to 0.990, *p* = 0.03, q = 0.07). NPC1L1-mediated LDL cholesterol showed a positive association with OA of the hip or knee (OR = 1.460, 95%CI: 1.127 to 1.890, *p* = 0.004, q = 0.033). Furthermore, LDLR-mediated LDL cholesterol demonstrated an association with OA of the hip or knee (OR = 0.882, 95%CI: 0.788 to 0.988, *p* = 0.03, q = 0.07) and hip osteoarthritis (OR = 0.867, 95%CI: 0.769 to 0.978, *p* = 0.02, q = 0.07).

**Conclusions:**

These findings provide preliminary evidence for the potential therapeutic use of lipid-lowering drugs in OA treatment. Further investigation is needed to validate these findings and explore the precise mechanisms underlying the observed associations.

## 1 Introduction

Osteoarthritis (OA) is a complex joint disorder with multifactorial etiology, characterized by the manifestation of joint pain, restricted joint mobility, and the potential for functional impairment. The pathogenesis of OA is characterized by synovitis, cartilage damage, excessive bone formation, and subchondral bone remodeling, often affecting multiple joints including the hand, hip, and knee [1, 2]. Presently, approximately 25% of the global population is afflicted with OA, with knee osteoarthritis (KOA) being the most prevalent form [3]. OA predominantly affects middle-aged and elderly women, and its etiology remains elusive, leading to a lack of effective treatments. In recent years, numerous investigations have suggested a potential association between the development of OA and metabolic syndrome (MetS), particularly involving lipid metabolism [4, 5]. With the shift in dietary patterns, hyperlipidemia has emerged as a significant health hazard among middle-aged and elderly individuals, contributing to cardiovascular disorders such as coronary heart disease, atherosclerosis, and hypertension. Growing evidence indicates the presence of abnormal lipid metabolism in OA patients, indicating that hyperlipidemia may serve as a risk factor for OA [6–8]. Lipid-lowering agents are established therapeutic interventions for hyperlipidemia, and multiple studies have demonstrated their antioxidant, antiproliferative, and anti-inflammatory properties, in addition to their lipid-lowering effects. Consequently, lipid-lowering drugs hold potential as a therapeutic option for arthritis treatment [9–11].

The effects of lipid-lowering drugs on OA have garnered significant attention among researchers. However, the findings thus far have been highly divergent, with some studies indicating a reduced risk of OA with lipid-lowering drugs, while others suggest no effect or even an increased risk of OA [12, 13]. These studies primarily rely on observational clinical data, which are inherently susceptible to confounding factors and have limitations in terms of feasibility, cost, and ethical considerations. Consequently, there is an urgent need to explore alternative research methods to investigate the effects of lipid-lowering drugs on OA.

Mendelian randomization(MR) analysis is an emerging epidemiological approach that utilizes comprehensive statistical data derived from genome-wide association studies (GWAS) to establish causal relationships between specific diseases and exposure factors, thereby identifying potential risk factors. By employing genetic variation as an instrumental variable for exposure factors, Mendelian randomization analysis overcomes the confounding factors commonly encountered in observational studies [14, 15]. In this study, we employed a drug-targeted Mendelian randomization approach to examine the association between genetically predicted lipid-modulating effects of commonly targeted lipid-lowering agents and the risk of OA. The aim is to provide a theoretical foundation for the use of lipid-lowering drugs in the treatment of OA.

## 2 Methods

All the studies incorporated in our analysis had received approval from their respective academic ethics review committees, and every participant had provided written informed consent. Ethical approval and consent to participate in the original GWASs were acquired from the appropriate review boards. It’s important to note that this current study constitutes a re-analysis of publicly accessible GWAS data, and therefore, no supplementary ethical approval was necessitated.

### 2.1 Experimental design

**Fig 1:**
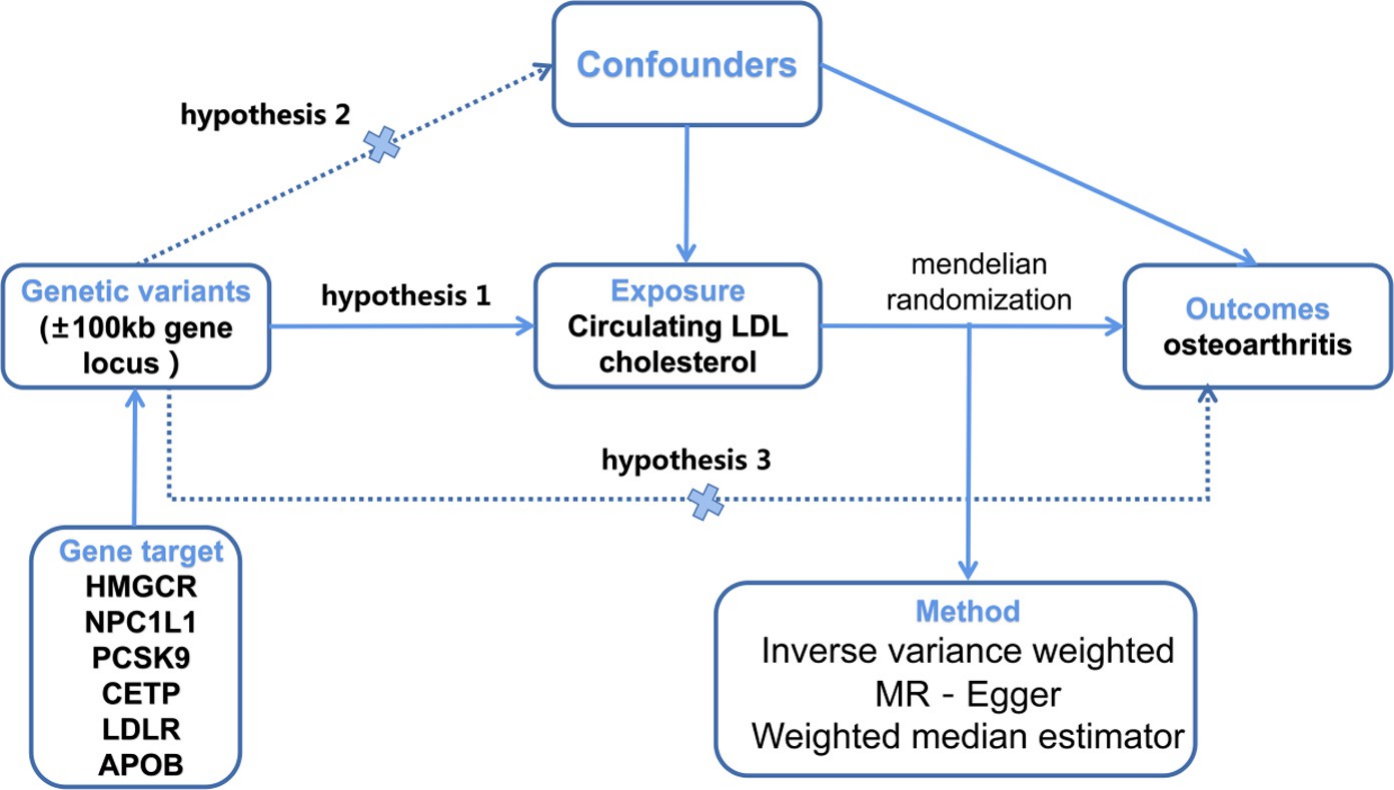
Diagram of the study design.

In the present investigation, Mendelian randomization (MR) analysis was employed to examine the potential causal relationship between lipid-lowering drugs and OA. Specifically, drug-target MR analysis was conducted to explore the association between genetically proxied inhibitors of 3-hydroxy-3-methyl glutaryl coenzyme A reductase (HMGCR, statin targets), Niemann-Pick C1-Like 1 (NPC1L1, ezetimibe target), proprotein convertase subtilisin/kexin type 9 (PCSK9, evolocumab and alirocumab targets), cholesterylester transfer protein (CETP, anacetrapib target), low-density lipoprotein receptor (LDLR), and apolipoprotein B (APOB, mipomersen target), and the risk of OA. To ensure the precision and reliability of the study, the design and analysis principles of a Mendelian randomization study were rigorously followed [16]. Firstly, the genetic variants used as instrumental variables (IVs) should exhibit no associations with confounding factors. Secondly, these genetic variants should not be correlated with confounding factors. Lastly, the genetic variants should influence the risk of the outcome solely through risk factors and not via alternative mechanisms. The MR analysis strictly adhered to the STROBE-MR statement (**S1 Checklist**) [17]. Since this study relied on existing publications and publicly available databases, no additional ethical approval or consent to participate was necessary. Further details regarding the design strategy can be found in **Fig 1**.

### 2.2 Data sources

The most recent summary statistics regarding lipid levels (n = 173,082) were obtained from the Global Lipid Genetics Consortium (GLGC) through the IEU GWAS database (https://gwas.mrcieu.ac.uk/terms/) at the University of Bristol, United Kingdom. These statistics were utilized for extracting genetic tools relevant to drug targeting, as previously described in the literature [18]. Genetic predictors associated with OA were sourced from the UK Biobank and arcOGEN resources. The UK Biobank dataset comprised 403,124 individuals for KOA (24,955 cases and 378,169 controls) [19], while the arcOGEN resource contained data from 393,873 individuals for hip osteoarthritis (15,704 cases and 378,169 controls) [19]. Researchers in previous studies have provided detailed explanations regarding the data employed for knee osteoarthritis and hip osteoarthritis [19]. The OA data utilized in this study were derived from European population studies. All datasets mentioned are publicly available in the GWAS dataset (https://gwas.mrcieu.ac.uk). Detailed information is given in **Table 1**.

**Table 1:** Characteristics of GWAS data for exposure and outcomes.

Trait	Variable type	sample size	Ancestry	Year
LDL	Exposure	173,082	European	2013
OA	Outcome	417,596	European	2019
KOA	Outcome	403,124	European	2019
HOA	Outcome	393,873	European	2019

### 2.3 Selection of instrumental variables

Within our study, we employed single nucleotide polymorphisms (SNPs) associated with lipid metabolism as surrogate targets for lipid-lowering drugs. Specifically, we identified three commonly utilized lipid-lowering drugs as the focus of our investigation and obtained information regarding their relevant drug protein targets from the DrugBank database [20, 21]. Following the methodology employed in prior studies, IVs were selected based on predetermined criteria. SNPs that exhibited significant associations with lipid profiles at the genome-wide level (P < 5.0 × 10^−8^) were included, provided that these SNPs were situated within ±100 kb of the specific gene region related to the drug target. To ensure the robustness of the instrumental variables, the selected SNPs were required to have an r^2^ value less than 0.3 and an effect allele frequency greater than 0.01 within the 100±kb range [22]. Additionally, all instrumental variables were screened for SNPs to avoid potential biases stemming from weak instrumental variables (F > 10). This was achieved by calculating the F statistic to ensure the strength of the instrumental variables [23]. Finally, given the demonstrated beneficial effects of lipid-lowering drugs on cardiovascular health, we conducted positive controls to validate the genetic proxies for the instrumental variables of lipid-lowering drugs. These positive controls aimed to further examine the association between the genetic proxies for lipid-lowering drugs and relevant outcomes, such as cardiovascular disease [24].

### 2.4 Mendelian randomization analysis

Within this study, we employed three MR methods: inverse-variance weighted (IVW) [25], weighted median (WM) [26], and MR-Egger [27]. The IVW method assumes the validity of all genetic variants as instrumental variables, calculates causal effect estimates for each individual instrumental variable using the ratio method, and aggregates these estimates. The main distinction between the MR-Egger method and the IVW method lies in the inclusion of an intercept term in the regression. The WM method utilizes the intermediate effects of all available genetic variants and derives estimates by weighting the inverse variance of each SNPs correlation with the outcome. In this study, the IVW method was selected as the preferred approach for estimating causal effects due to its higher test efficacy compared to the other two MR methods [28].

### 2.5 Horizontal pleiotropy analysis and heterogeneity tests

The intercept term in the MR-Egger regression method is commonly employed to evaluate the presence of horizontal pleiotropy. If the intercept term is close to zero, it indicates the absence of horizontal pleiotropy within the study [26]. Additionally, Cochran’s Q statistic was employed to assess the presence of heterogeneity. If the p-value of the Cochran’s Q test is statistically significant, it indicates heterogeneous results [29]. Furthermore, a leave-one-out analysis was conducted to ensure the reliability of the overall effect. This analysis examines the effect of excluding individual SNPs on the overall causal estimates, thus evaluating the robustness of the findings [30].

### 2.6 Statistical analysis

The results of this study are presented as odds ratios (OR) with corresponding 95% confidence intervals (CI). We conducted 18 tests and employed the Benjamini-Hochberg False Discovery Rate (FDR) procedure, setting a threshold of q value< 0.1 for FDR correction [31]. All statistical analyses were performed using R version 4.1.0. The "TwoSampleMR", "qvalue" and "MRPRESSO" software packages were employed for the analysis.

## 3 Result

### 3.1 Genetic instruments selection

We procured an array of genetic variants associated with LDL cholesterol levels from the GWAS summary data collated by the Global Lipids Genetics Consortium. From the dataset, we extracted 7, 12, 3, 9, 8, and 9 SNPs associated with the HMGCR, PCSK9, NPC1L1, APOB, CETP, and LDLR genes, respectively. These SNPs were selected based on rigorous criteria to ensure the robustness of our instrumental variables. Further details are provided in **S1 Table in S1 Data**. Our study, with F-statistics exceeding 10 for all instrumental variants, illustrates a successful mitigation of bias that could result from weak instrumental variables. Using LDL cholesterol GWAS-proposed instruments (**S2 Table in S1 Data**) for our positive control study, we identified significant associations between drug exposure and coronary heart disease prevalence, affirming the efficacy of the genetic instruments we selected.

### 3.2 Mendelian randomization analysis

**Fig 2:**
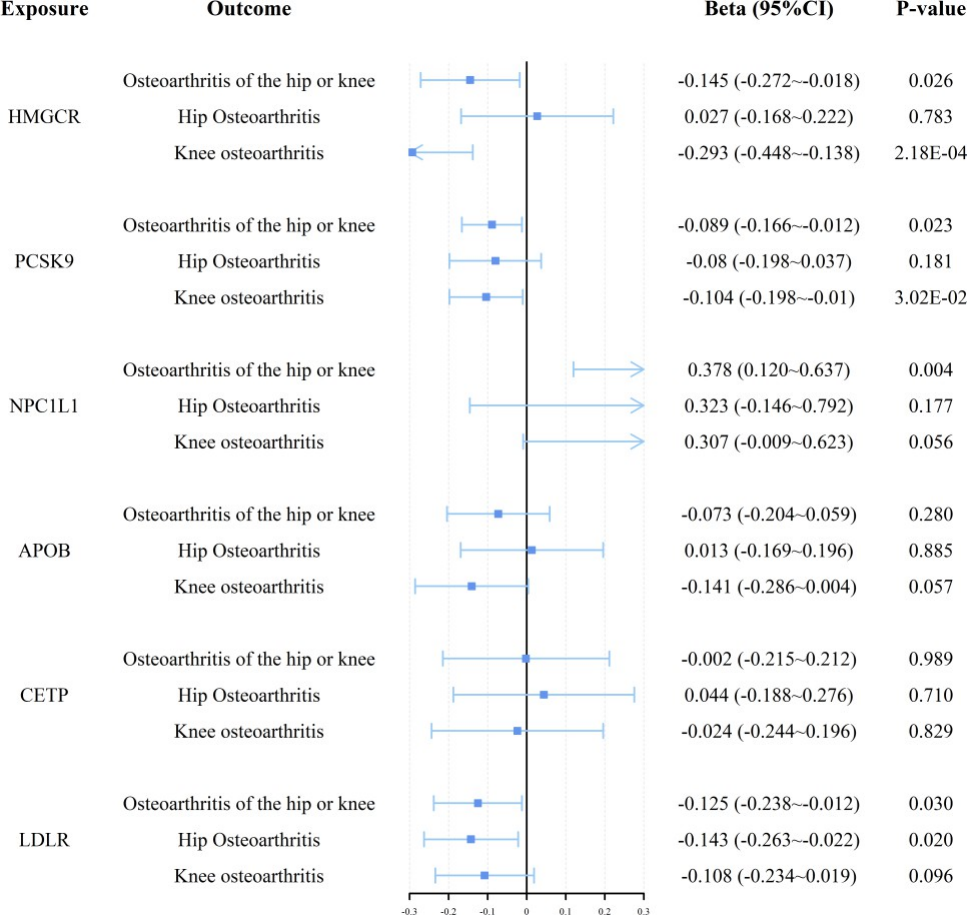
Inverse-variance weighted Mendelian randomization (IVW-MR) association between low- density lipoprotein (LDL) cholesterol mediated by gene 3-hydroxy-3-methylglutaryl-coenzyme A reductase(HMGCR), Niemann-Pick C1-Like 1 (NPC1L1, proprotein convertase subtilisin/kexin type 9 (PCSK9), cholesterylester transfer protein (CETP), low-density lipoprotein receptor (LDLR,), or apolipoprotein B (APOB) and OA outcomes. IVW- MR method was used to assess the association.

Through the current MR analysis, we identified multiple significant associations between genetically proxied lipid-lowering drug targets and OA risk, as detailed in **Fig 2** and **S3 Table in S1 Data**. Notably, HMGCR-mediated LDL cholesterol, a target for statins, demonstrated a suggestive association with both overall OA of the hip or knee (OR = 0.865, 95%CI: 0.762 to 0.983, *p* = 0.026, q = 0.07) and knee osteoarthritis specifically (OR = 0.746, 95%CI: 0.639 to 0.871, *p* = 2.180×10^−4^, q = 0.004). PCSK9-mediated LDL cholesterol, targeted by drugs such as evolocumab and alirocumab, also showed a suggestive relationship with OA of the hip or knee (OR = 0.915, 95%CI: 0.847 to 0.988, *p* = 0.023, q = 0.07) and knee osteoarthritis (OR = 0.901, 95%CI: 0.821 to 0.990, *p* = 0.03, q = 0.07). NPC1L1-mediated LDL cholesterol, the target of ezetimibe, presented a suggestive positive association with OA of the hip or knee (OR = 1.460, 95%CI: 1.127 to 1.890, *p* = 0.004, q = 0.033). Furthermore, LDLR-mediated LDL cholesterol, also demonstrated a suggestive association with OA of the hip or knee (OR = 0.882, 95%CI: 0.788 to 0.988, *p* = 0.03, q = 0.07) and hip osteoarthritis (OR = 0.867, 95%CI: 0.769 to 0.978, p = 0.02, q = 0.07).

### 3.3 Sensitivity analysis

We implemented Cochran’s Q test and assessed the MR-Egger intercept to scrutinize the possible influence of heterogeneity and horizontal pleiotropy on our estimates. In all other analyses outside of the LDLR-mediated LDL cholesterol examination, these elements showed no signs of impacting our calculations (**S3 Table in S1 Data**). Moreover, our leave-one-out sensitivity checks indicated that no significant changes in effect estimates arose when any individual SNP was excluded (refer to **S1-S18 Figs in S1 File**). This reinforces that our findings are not tied to the impact of any particular SNP. When we specifically investigated the causal relationships between LDLR-mediated LDL cholesterol and OA of the hip or knee, as well as knee OA, we observed instances of heterogeneity and pleiotropy (Cochran Q test *p* value = 0.034; MR-Egger intercept *p* values = 0.030 and 0.039 respectively). Despite these observations in the LDLR analysis, MR-PRESSO detected no outliers, and the Phenoscanner tool identified no multi-effect SNPs.

## 4 Discussion

To our current knowledge, this study represents the inaugural endeavor to investigate the causal association between drug target-mediated lipid levels and OA, encompassing both knee and hip arthritis. We employed a Mendelian randomization approach to ensure the reliability of our findings by leveraging genetic variants to control for potential confounding factors. A two-sample Mendelian randomization study was conducted utilizing publicly available GWAS datasets. The results presented in **Fig 2** and **S3 Table in S1 Data** indicate a suggested correlation between lipid-lowering drugs and OA. Furthermore, our robustness analysis demonstrated the consistency and stability of our results.

Given the increasing prevalence of OA, repurposing existing drugs offers advantages in terms of economic and time costs compared to developing new drugs. Recent studies have highlighted the significant role of lipid metabolism in the pathogenesis of OA, leading to increased interest in repurposing lipid-lowering drugs for OA management. Statins, as widely used drugs in clinical practice, have demonstrated preventive effects against cardiovascular disease and have also been identified for their anti-inflammatory and immunomodulatory properties, making them potential candidates for OA treatment. Clinical investigations have shown that lipid-lowering drugs can decelerate the progression of OA [11, 32–34]. Notably, a large retrospective cohort study involving 2,921 cases indicated that statin use reduced the overall progression of knee osteoarthritis by more than 50% compared to non-statin users, although no similar effect was observed in hip osteoarthritis [35]. Conversely, a study from the USA presented contrasting results, suggesting that statin use did not lead to improvements in knee pain, function, or structural progression over a 4-year follow-up period among 2,207 patients [36]. Additionally, statin use has been associated with an increased risk of OA and arthropathy [37], and another study reported an elevated risk of new-onset hip osteoarthritis among 5,674 patients during an 8-year follow-up period [38]. These conflicting findings disrupt clinical practice and can be attributed to variations in sample selection, data collection, analysis methods, population characteristics, age, and the inherent confounding and reverse causality issues associated with retrospective observational studies, which hinder causal inferences.

Mendelian randomization studies, as a novel genetic epidemiological approach, can overcome the limitations of traditional observational studies. In this study, we utilized genetic variants related to HMGCR-mediated LDL cholesterol as instruments to investigate statin exposure as a proxy. The results indicated that HMGCR inhibition reduces the risk of knee osteoarthritis but not hip osteoarthritis. Despite the need for additional robust evidence, these findings provide valuable causal insights. Existing studies suggest that inflammatory factors such as IL-1β, IL-8, and TNF-α play pivotal roles in the pathogenesis of OA. IL-1β and TNF-α have been found to synergistically promote the expression of osteoclast differentiation factor (RANKL) in bone marrow osteoblast stromal cells, indirectly inducing osteoclastogenesis [39]. The inflammatory response in OA leads to the overexpression of pro-inflammatory factors like IL-1β, IL-8, and TNF-α in synovial membranes, contributing to angiogenesis [40]. In chondrocytes, IL-1β and IL-8 can mediate the production of MMP-1, MMP-3, and MMP-9, which subsequently promote the degradation of type II collagen, ultimately leading to cartilage and joint damage. Pharmacological studies have demonstrated that statins reduce the production of IL-6 and IL-1β, providing immunomodulatory effects through T cell activation and inhibiting the IFNγ-induced expression of the class II major histocompatibility complex [41]. Therefore, statins may ameliorate the course of OA by modulating inflammatory factors. Furthermore, statins have shown protective effects on cartilage. For instance, an in vitro study using an IL-1β-induced rat articular exosome culture model revealed the protective effect of atorvastatin on cartilage against IL-1β treatment [42]. Moreover, oxidative stress, considered a contributing factor to osteoarthritis pain and cartilage degeneration, was addressed in a study using monosodium p-iodoacetate (MIA)-treated osteoarthritic rats. The findings demonstrated that atorvastatin reduced MIA-induced osteoarthritis symptoms by inhibiting oxidative stress and protecting articular cartilage from damage [43]. These studies indicate the therapeutic potential of statins in OA management, providing a theoretical foundation for their utilization.

Ezetimibe is extensively utilized as a cholesterol absorption inhibitor in clinical therapy for various ailments. Multiple investigations have demonstrated that ezetimibe possesses inhibitory properties against the inflammatory response across diverse diseases [44, 45], including rheumatoid arthritis [46, 47]. Recent research has indicated that ezetimibe hinders the absorption of dietary and biliary cholesterol in the intestines, potentially resulting in the accumulation of triglycerides (TGs) or cholesterol within the gastrointestinal tract. A mounting body of evidence suggests that the gut microbiota significantly impacts drug metabolism, influencing both their efficacy and side effects. The intestinal flora is comprised of bacteria, archaea, and eukaryotic organisms that carry out critical physiological functions within the host. These functions encompass the modulation of host immunity and the prevention of pathogen intrusion [48]. Dysregulation of the intestinal flora can lead to intestinal barrier dysfunction, heightened intestinal permeability, and the release of toxic bacterial metabolites into the bloodstream, thereby fostering the development of systemic low-grade inflammation [49]. Furthermore, the imbalance of the intestinal flora triggers the activation of the innate immune system, leading to an upsurge in pro-inflammatory factors that can affect the joints [50]. Researchers have uncovered that ezetimibe can influence the composition and equilibrium of the intestinal flora. Specifically, ezetimibe-induced alterations include a reduction in low-abundance bacteria such as Aspergillus and Desulfovibrio, along with an increase in Anaplasma spp. These changes are accompanied by substantial modifications in the intestinal microbiota and short-chain fatty acids (SCFAs) [51]. The disruption of tight junctions between intestinal epithelial cells can occur due to an imbalanced intestinal flora, permitting the entry of intestinal luminal bacteria and lipopolysaccharides (LPS) into the systemic circulation via paracellular transport. In this process, bacterial peptide glycans (PGN) and LPS serve as ligands that bind to pattern recognition receptors, subsequently eliciting pro-inflammatory responses [52]. PGN binds to Toll-like receptor 2 and nucleotide-binding and oligomerization domain-like receptor (NLR), ultimately inducing the expression of matrix metalloproteinases (MMPs) [53, 54]. Moreover, LPS activates macrophages in the innate immune system to transition into M1-type macrophages and form complexes with Toll-like receptor 4. This, in turn, prompts the synthesis of pro-inflammatory factors, including interleukin-1β (IL-1β), tumor necrosis factor (TNF), MMPs, and free radicals, leading to pronounced secondary inflammation in joint tissues and throughout the body [55, 56]. These findings collectively suggest that ezetimibe may disrupt the balance of the intestinal flora, potentially exacerbating osteoarthritis symptoms, where the intestinal flora serves as a pivotal mediating factor. However, it is important to note that clinical studies have primarily focused on the therapeutic effects of statins, with limited investigations on other lipid-lowering drugs such as PCSK9 inhibitors (e.g., alirocumab and evolocumab), CETP inhibitors (e.g., anacetrapib) for OA. Future clinical studies should explore these drugs in greater detail.

While this study adopted a Mendelian randomization approach to infer the association between lipid-lowering drugs and OA, providing more robust results than traditional case-control studies, certain limitations need to be acknowledged. First, the majority of GWAS data predominantly focus on European populations, resulting in limited representation of Asian populations. Consequently, genetic differences may exist between the European population chosen for this study and other populations. Second, although MR analysis can investigate causal relationships, it does not elucidate specific biological mechanisms. Third, the instrumental variable selection criteria employed in MR analysis are ideal, and future studies could employ more stringent criteria to enhance result reliability. Fourth, the GWAS data used in this analysis lacked stratified analysis based on factors such as sex, age, and disease duration, limiting the ability to study specific details. Therefore, future studies with larger sample sizes, increased instrumental variables, and comprehensive stratified analyses are necessary to enhance the reliability of the results and explore further avenues of research.

In conclusion, our study employed a Mendelian randomization approach to investigate the potential causal relationship between lipid-lowering drugs and OA. Our findings indicate that lipid-lowering drugs could potentially serve as a protective factor for arthritis, suggesting their potential as therapeutic agents in the management of OA. However, due to the intricate nature of the relationship between lipid-lowering drugs and OA, further clinical investigations are warranted to comprehensively elucidate the underlying mechanisms and ascertain their clinical implications. Consequently, our study serves as a foundational stepping stone for future research endeavors in this significant realm of inquiry.

## Supporting information

S1 File (DOCX)

S1 Data (XLSX)

S1 Checklist STROBE-MR checklist of recommended items to address in reports of Mendelian randomization studies^1 2^. (DOC)
