# Supplementary material for: Association of lipid-lowering drugs with osteoarthritis outcomes from a drug-target Mendelian randomization study
Source: PLoS One. 2024 Feb 28;19(2):e0293960. doi: 10.1371/journal.pone.0293960 (PMC10901306; doi:10.1371/journal.pone.0293960)
Supplement: S1 File — (DOCX) [file pone.0293960.s001.docx]

**
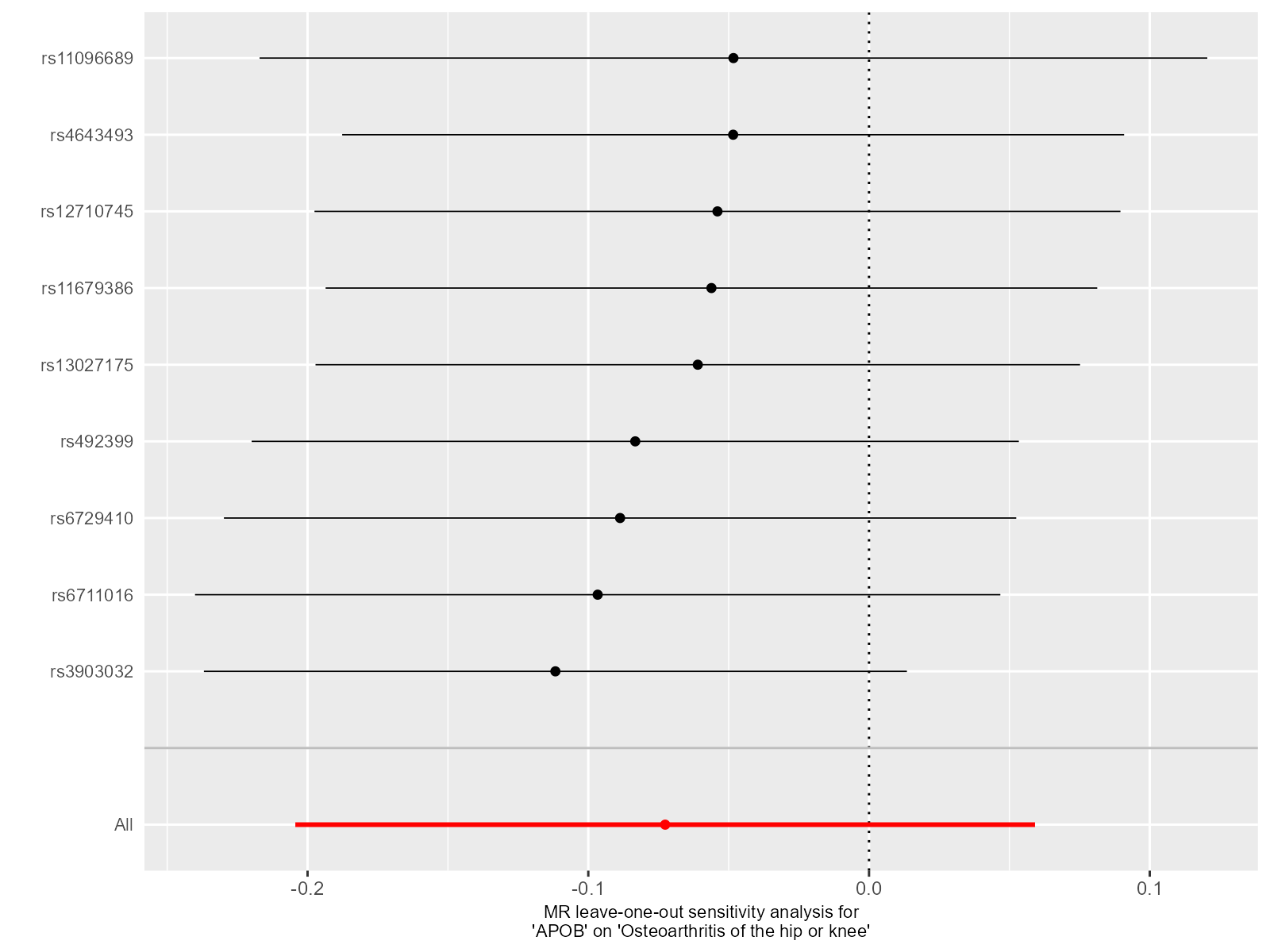
Supplementary Figure 1. Leave-one-out plot for sensitivity analysis of single SNP effect on** “**APOB”-to-“osteoarthritis of the hip or knee” UVMR results.**

Leave-one-out plot using IVW method by sequentially re-evaluating the causal estimate after discarding one SNP at a time, which helps determine whether the overall effect is driven by the specific genetic variant. The black point denotes the causal effect estimate after discarding a certain SNP, and the black line signifies the 95% CI of estimate. The red point symbolizes the causal effect estimate, and the red line indicates the 95% CI of the estimate.

**
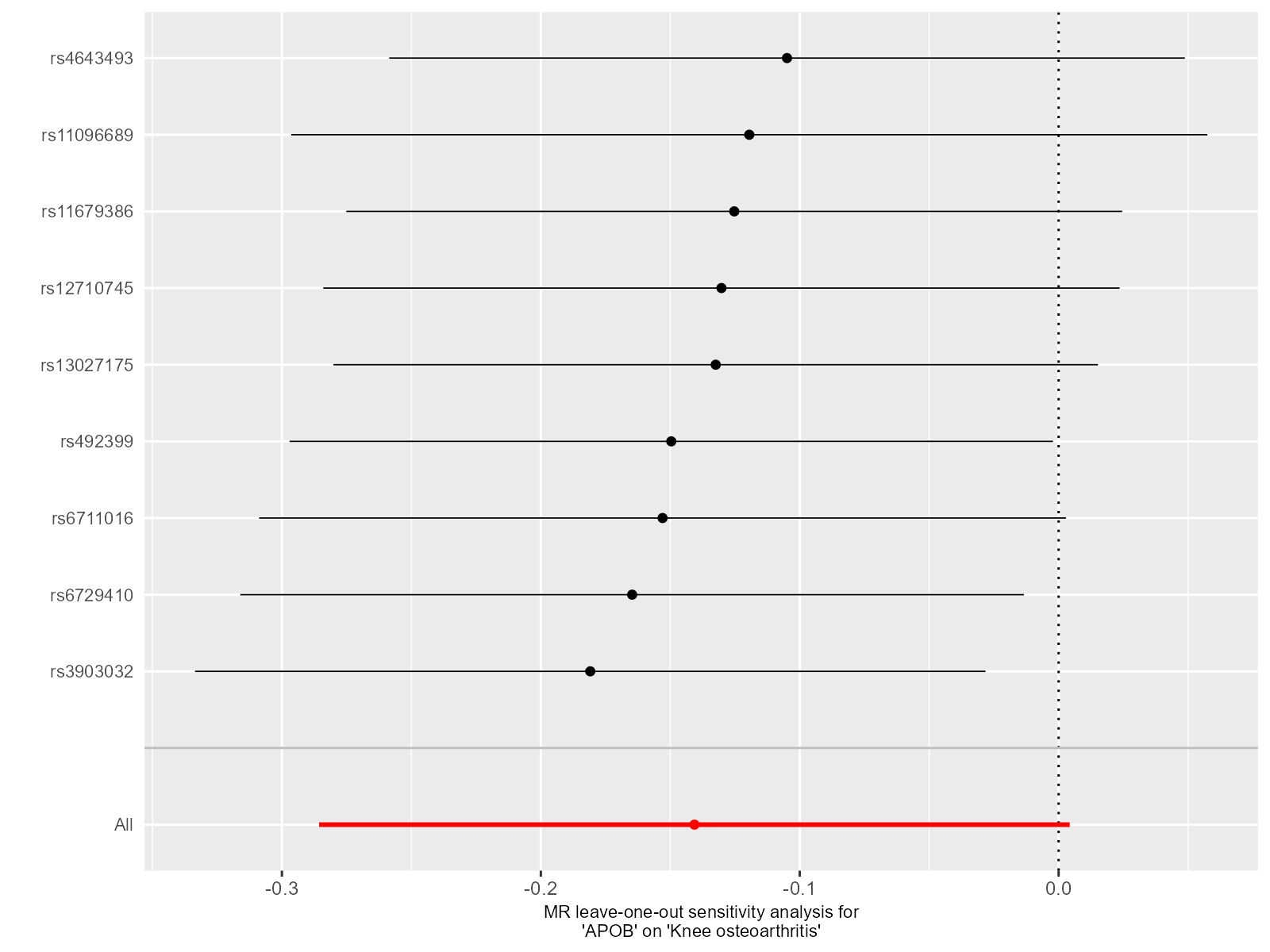
Supplementary Figure 2. Leave-one-out plot for sensitivity analysis of single SNP effect on** “**APOB”-to-“knee osteoarthritis” UVMR results.**

Leave-one-out plot using IVW method by sequentially re-evaluating the causal estimate after discarding one SNP at a time, which helps determine whether the overall effect is driven by the specific genetic variant. The black point denotes the causal effect estimate after discarding a certain SNP, and the black line signifies the 95% CI of estimate. The red point symbolizes the causal effect estimate, and the red line indicates the 95% CI of the estimate.

**
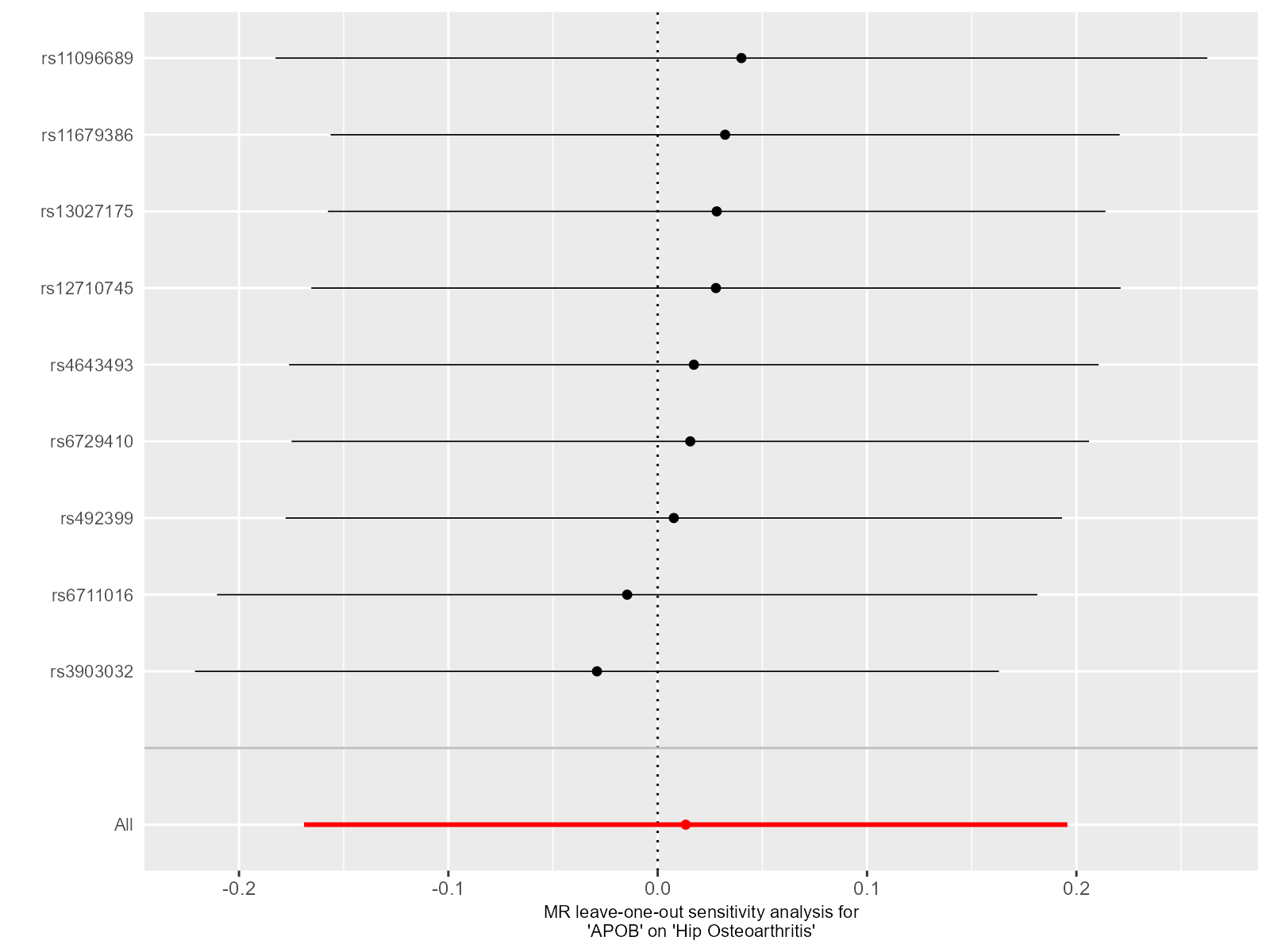
Supplementary Figure 3. Leave-one-out plot for sensitivity analysis of single SNP effect on** “**APOB”-to-“hip osteoarthritis” UVMR results.**

Leave-one-out plot using IVW method by sequentially re-evaluating the causal estimate after discarding one SNP at a time, which helps determine whether the overall effect is driven by the specific genetic variant. The black point denotes the causal effect estimate after discarding a certain SNP, and the black line signifies the 95% CI of estimate. The red point symbolizes the causal effect estimate, and the red line indicates the 95% CI of the estimate.

**
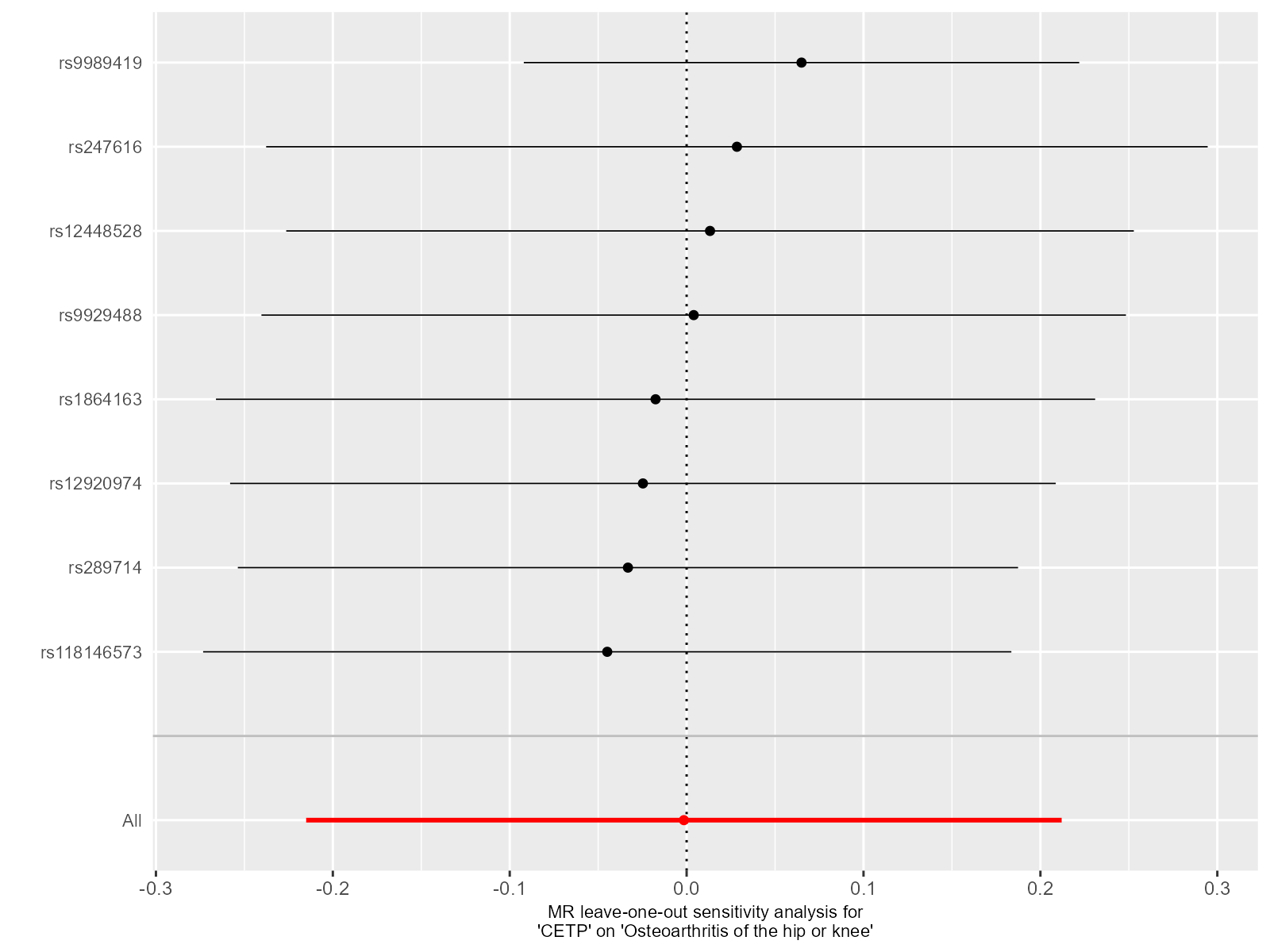
Supplementary Figure 4. Leave-one-out plot for sensitivity analysis of single SNP effect on** “**CETP”-to-“osteoarthritis of the hip or knee” UVMR results.**

Leave-one-out plot using IVW method by sequentially re-evaluating the causal estimate after discarding one SNP at a time, which helps determine whether the overall effect is driven by the specific genetic variant. The black point denotes the causal effect estimate after discarding a certain SNP, and the black line signifies the 95% CI of estimate. The red point symbolizes the causal effect estimate, and the red line indicates the 95% CI of the estimate.

**
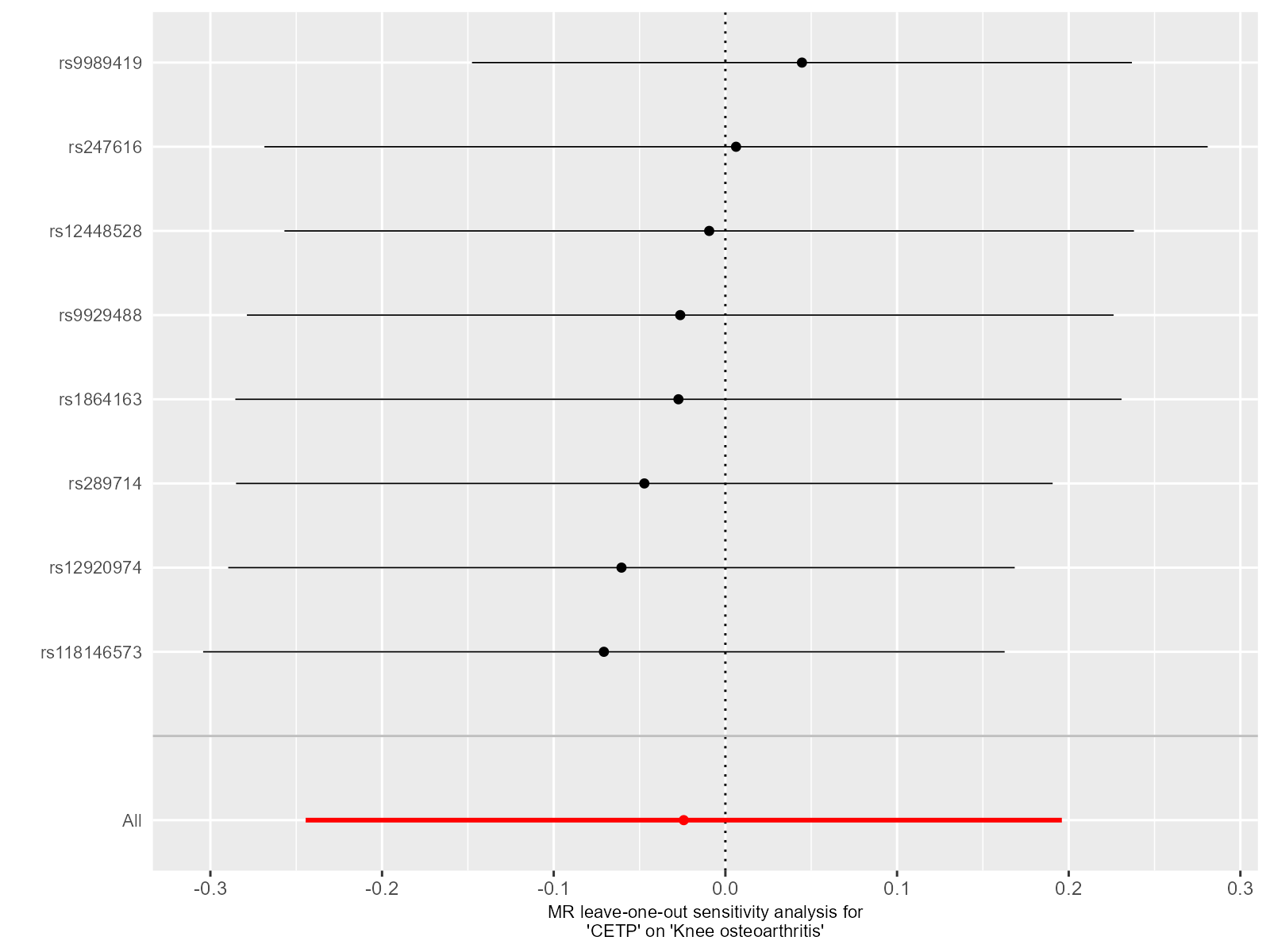
Supplementary Figure 5. Leave-one-out plot for sensitivity analysis of single SNP effect on** “**CETP”-to-“knee osteoarthritis” UVMR results.**

Leave-one-out plot using IVW method by sequentially re-evaluating the causal estimate after discarding one SNP at a time, which helps determine whether the overall effect is driven by the specific genetic variant. The black point denotes the causal effect estimate after discarding a certain SNP, and the black line signifies the 95% CI of estimate. The red point symbolizes the causal effect estimate, and the red line indicates the 95% CI of the estimate.

**
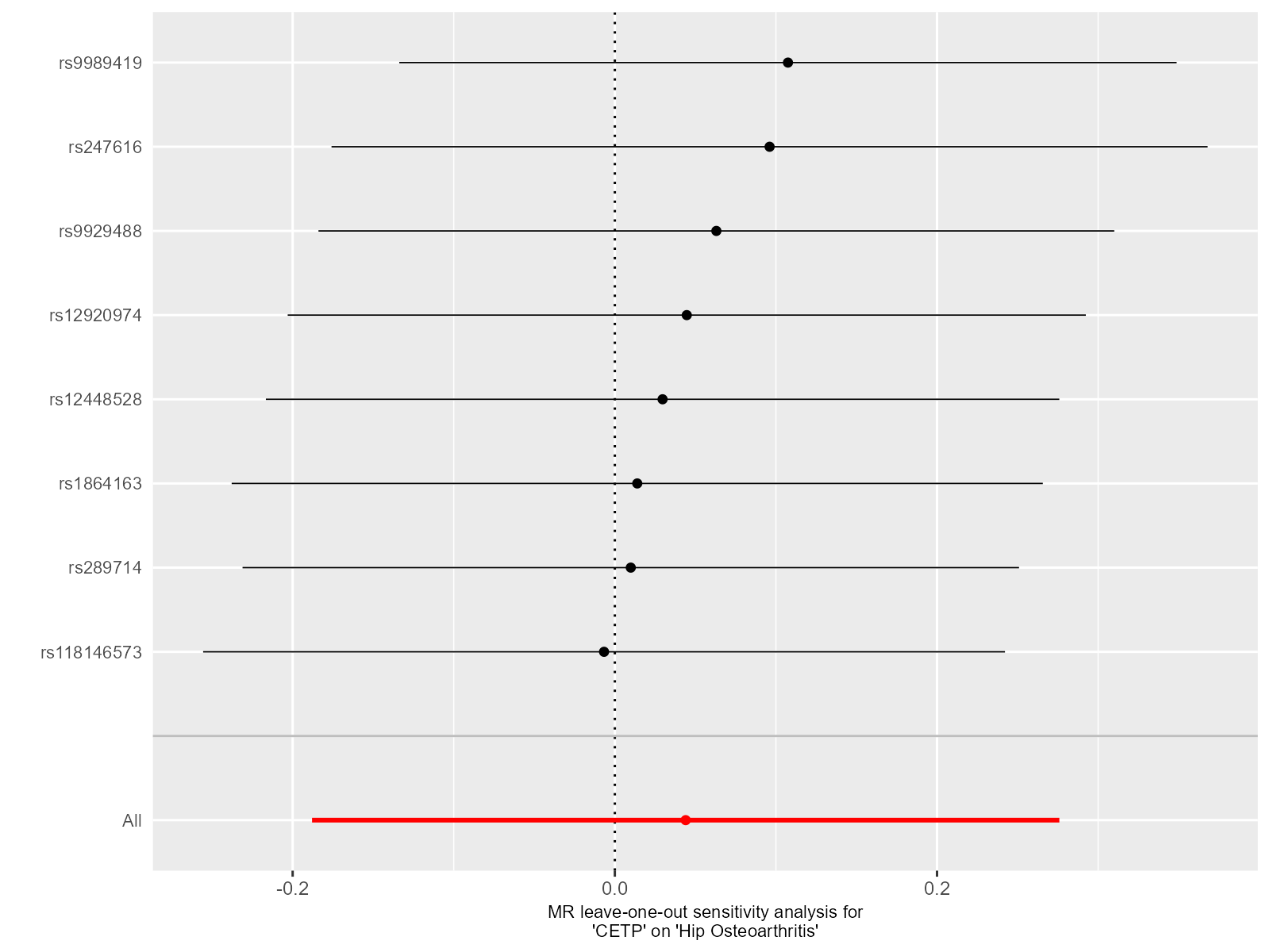
Supplementary Figure 6. Leave-one-out plot for sensitivity analysis of single SNP effect on** “**CETP”-to-“hip osteoarthritis” UVMR results.**

Leave-one-out plot using IVW method by sequentially re-evaluating the causal estimate after discarding one SNP at a time, which helps determine whether the overall effect is driven by the specific genetic variant. The black point denotes the causal effect estimate after discarding a certain SNP, and the black line signifies the 95% CI of estimate. The red point symbolizes the causal effect estimate, and the red line indicates the 95% CI of the estimate.

**
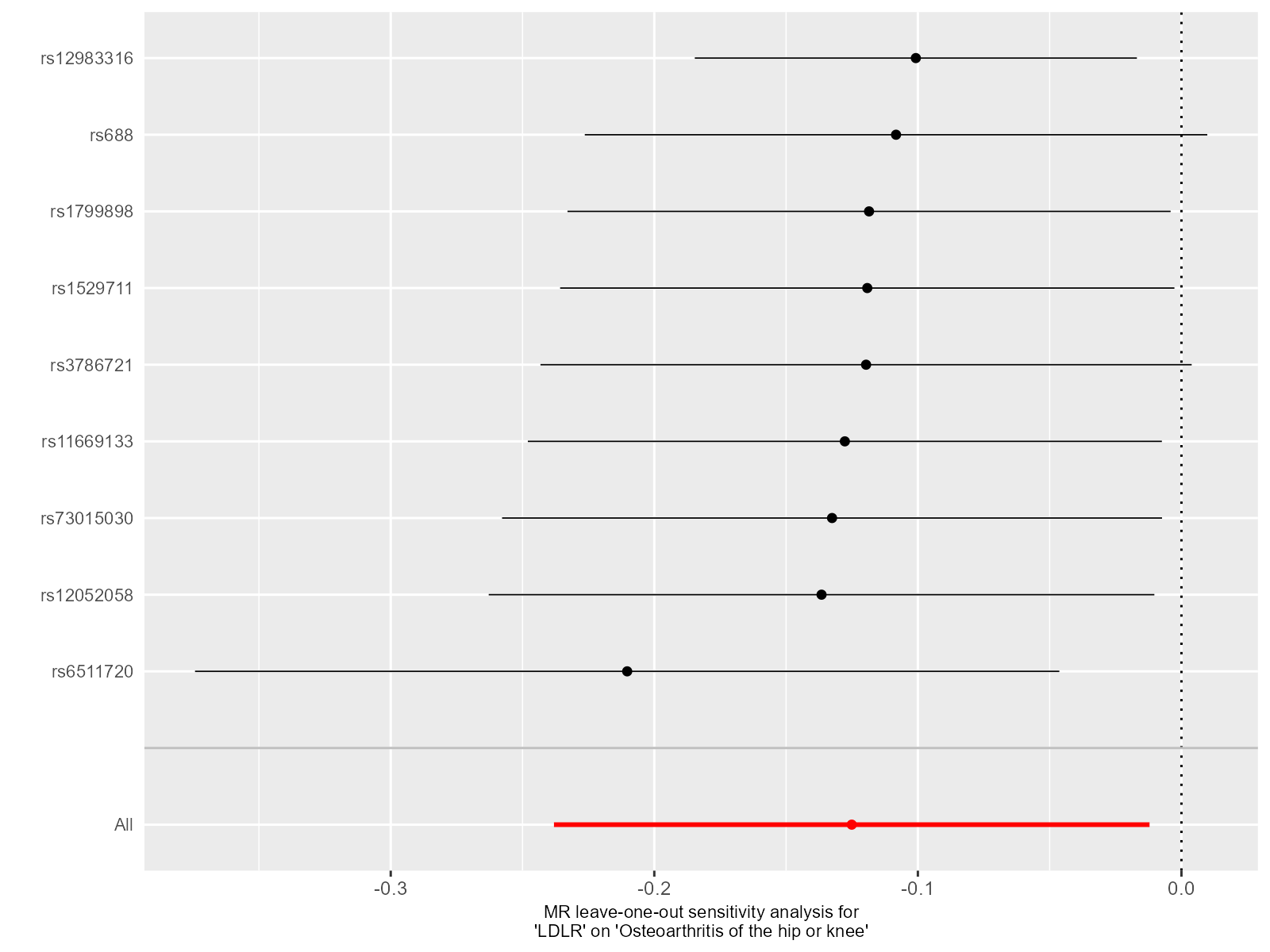
Supplementary Figure 7. Leave-one-out plot for sensitivity analysis of single SNP effect on** “**LDLR”-to-“osteoarthritis of the hip or knee” UVMR results.**

Leave-one-out plot using IVW method by sequentially re-evaluating the causal estimate after discarding one SNP at a time, which helps determine whether the overall effect is driven by the specific genetic variant. The black point denotes the causal effect estimate after discarding a certain SNP, and the black line signifies the 95% CI of estimate. The red point symbolizes the causal effect estimate, and the red line indicates the 95% CI of the estimate.

**
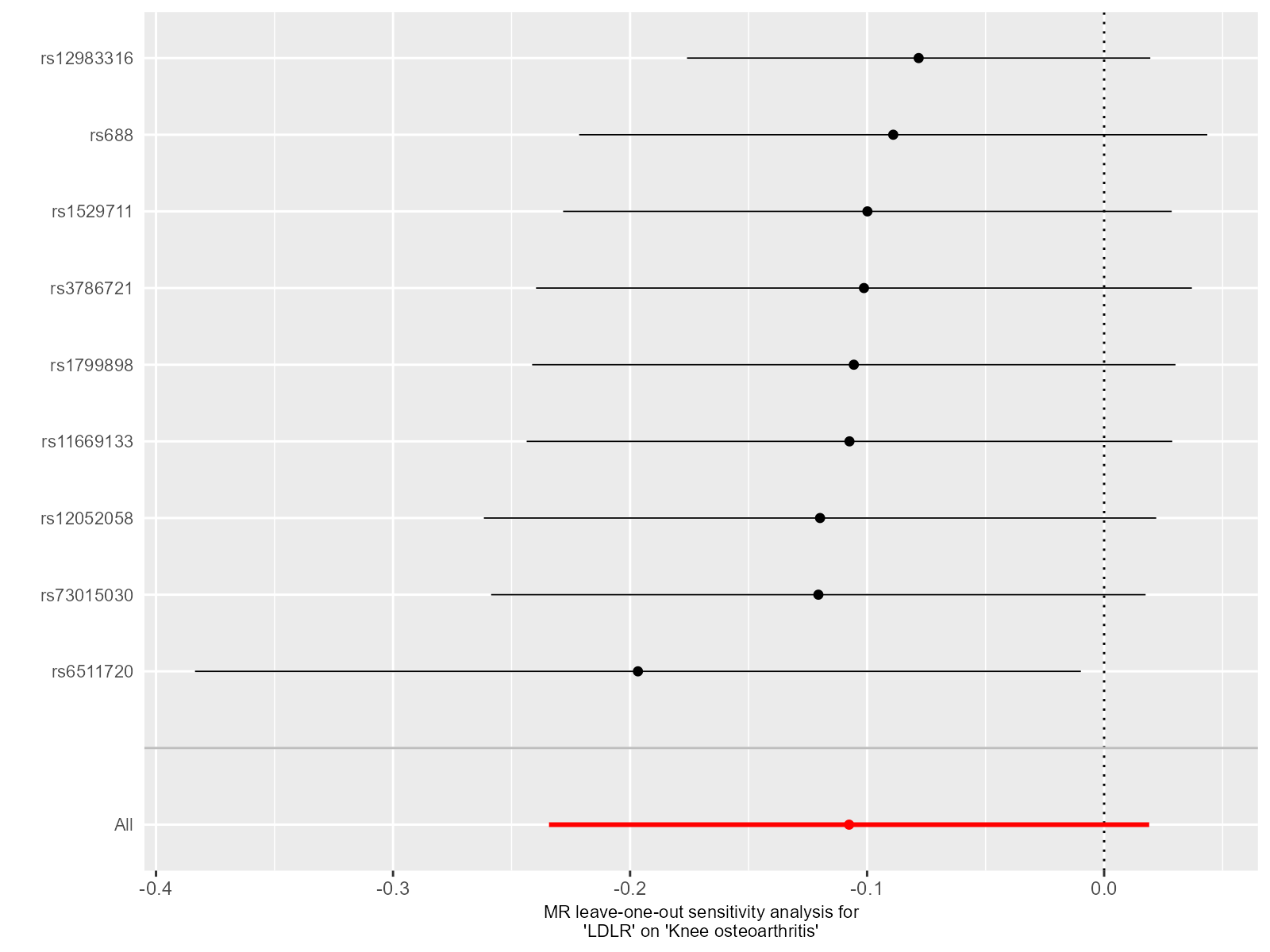
Supplementary Figure 8. Leave-one-out plot for sensitivity analysis of single SNP effect on** “**LDLR”-to-“knee osteoarthritis” UVMR results.**

Leave-one-out plot using IVW method by sequentially re-evaluating the causal estimate after discarding one SNP at a time, which helps determine whether the overall effect is driven by the specific genetic variant. The black point denotes the causal effect estimate after discarding a certain SNP, and the black line signifies the 95% CI of estimate. The red point symbolizes the causal effect estimate, and the red line indicates the 95% CI of the estimate.

**
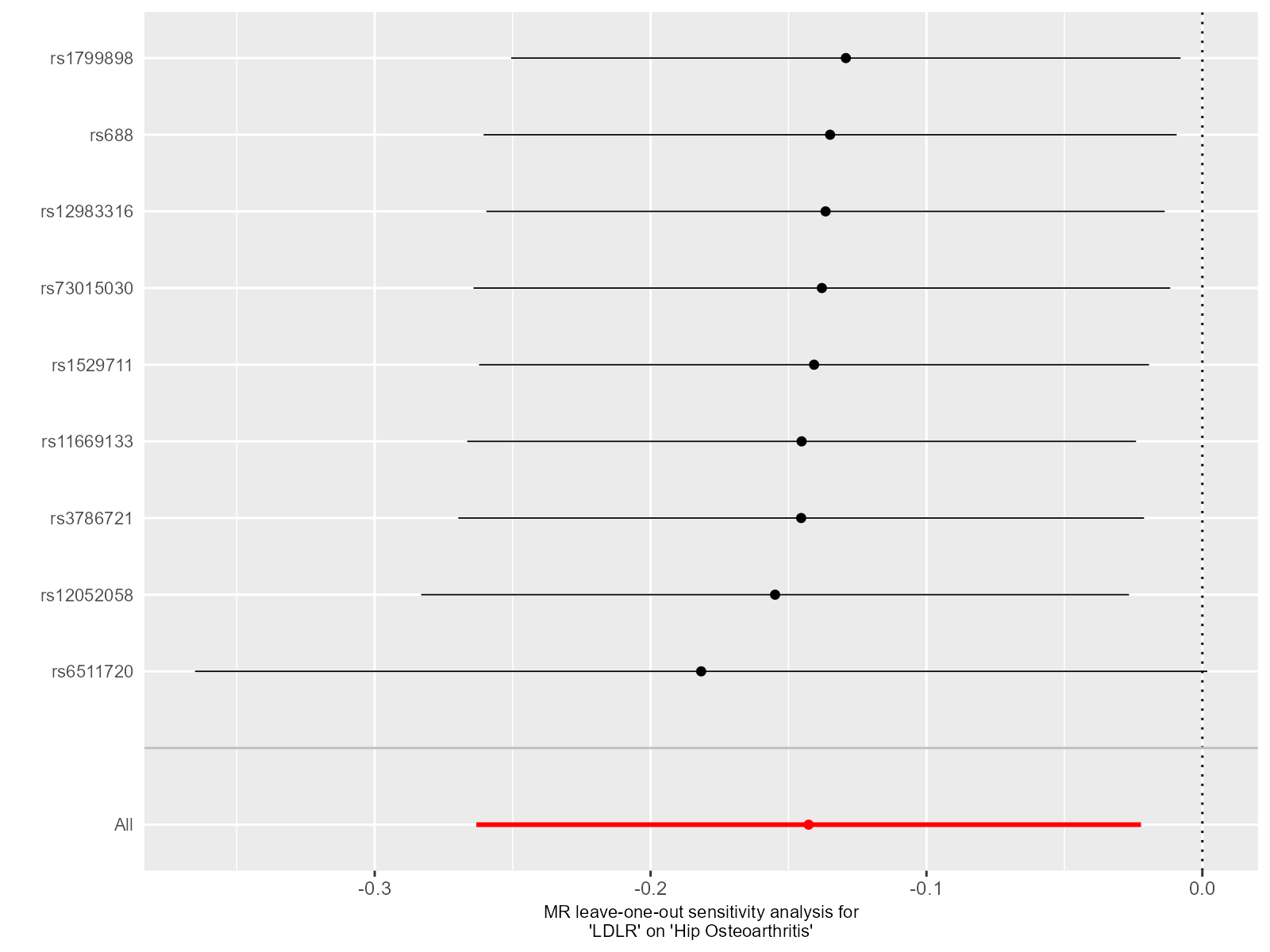
Supplementary Figure 9. Leave-one-out plot for sensitivity analysis of single SNP effect on** “**LDLR”-to-“hip osteoarthritis” UVMR results.**

Leave-one-out plot using IVW method by sequentially re-evaluating the causal estimate after discarding one SNP at a time, which helps determine whether the overall effect is driven by the specific genetic variant. The black point denotes the causal effect estimate after discarding a certain SNP, and the black line signifies the 95% CI of estimate. The red point symbolizes the causal effect estimate, and the red line indicates the 95% CI of the estimate.

**
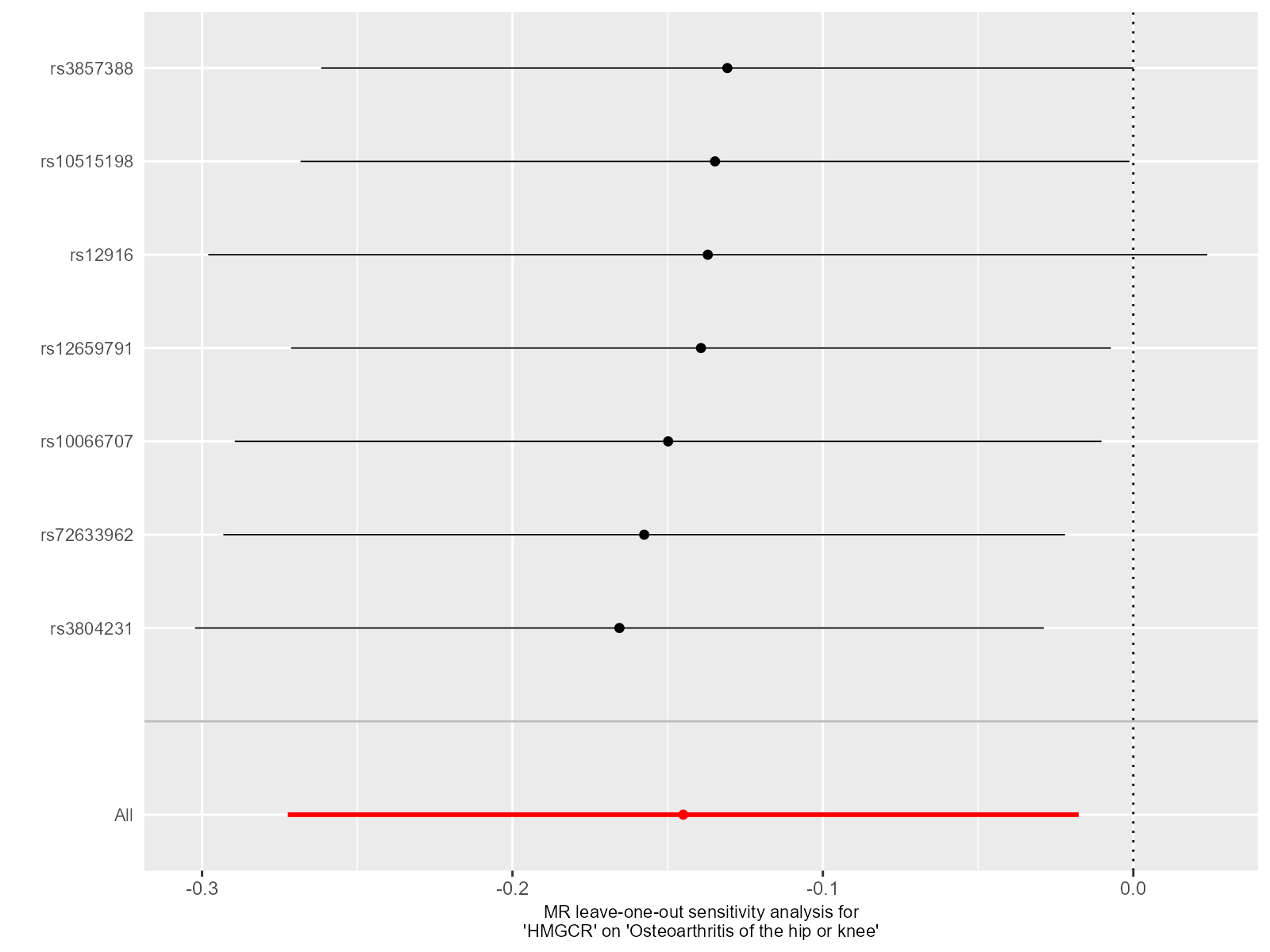
Supplementary Figure 10. Leave-one-out plot for sensitivity analysis of single SNP effect on** “**HMGCR”-to-“osteoarthritis of the hip or knee” UVMR results.**

Leave-one-out plot using IVW method by sequentially re-evaluating the causal estimate after discarding one SNP at a time, which helps determine whether the overall effect is driven by the specific genetic variant. The black point denotes the causal effect estimate after discarding a certain SNP, and the black line signifies the 95% CI of estimate. The red point symbolizes the causal effect estimate, and the red line indicates the 95% CI of the estimate.

**
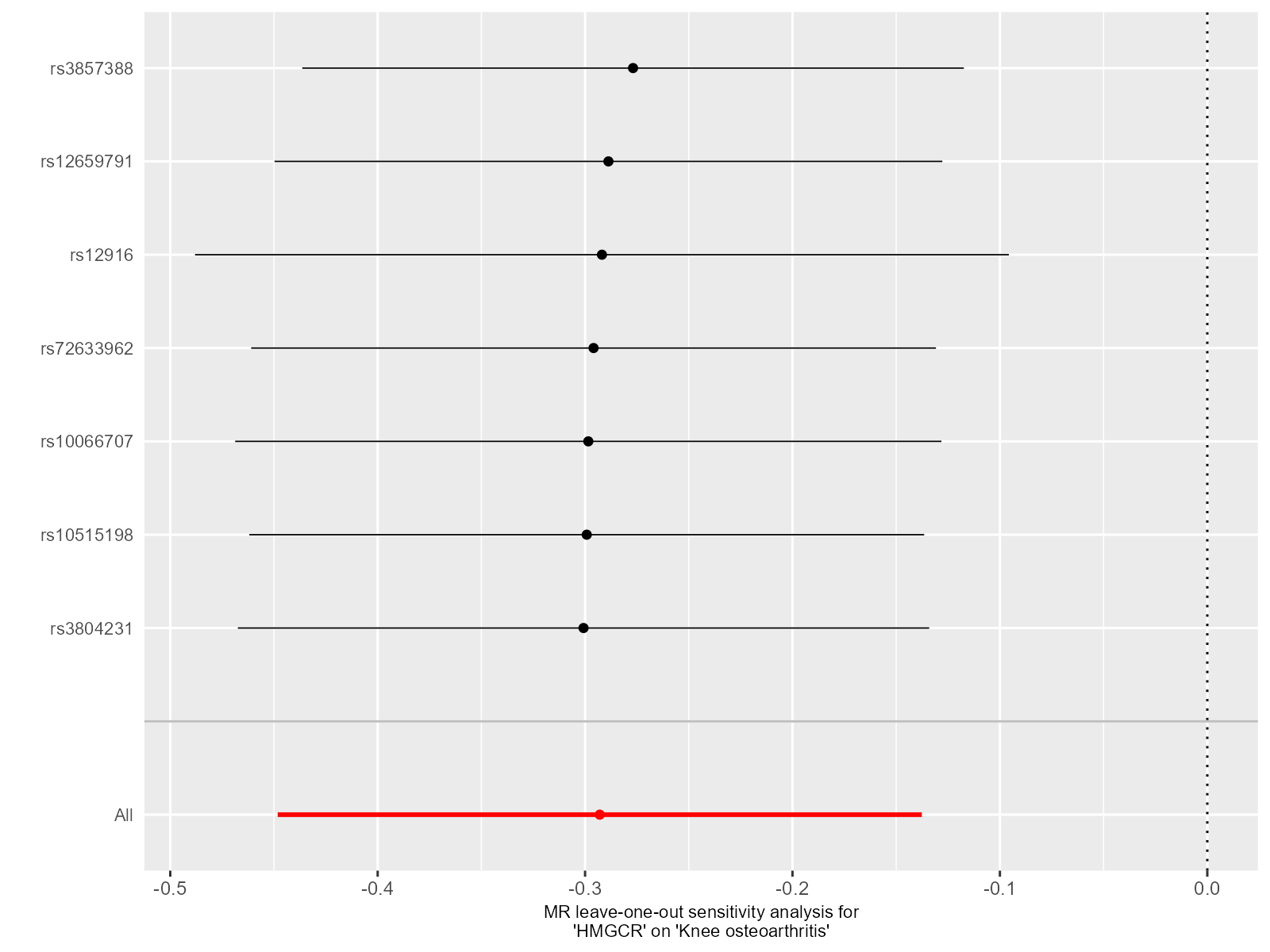
Supplementary Figure 11. Leave-one-out plot for sensitivity analysis of single SNP effect on** “**HMGCR”-to-“knee osteoarthritis” UVMR results.**

Leave-one-out plot using IVW method by sequentially re-evaluating the causal estimate after discarding one SNP at a time, which helps determine whether the overall effect is driven by the specific genetic variant. The black point denotes the causal effect estimate after discarding a certain SNP, and the black line signifies the 95% CI of estimate. The red point symbolizes the causal effect estimate, and the red line indicates the 95% CI of the estimate.

**
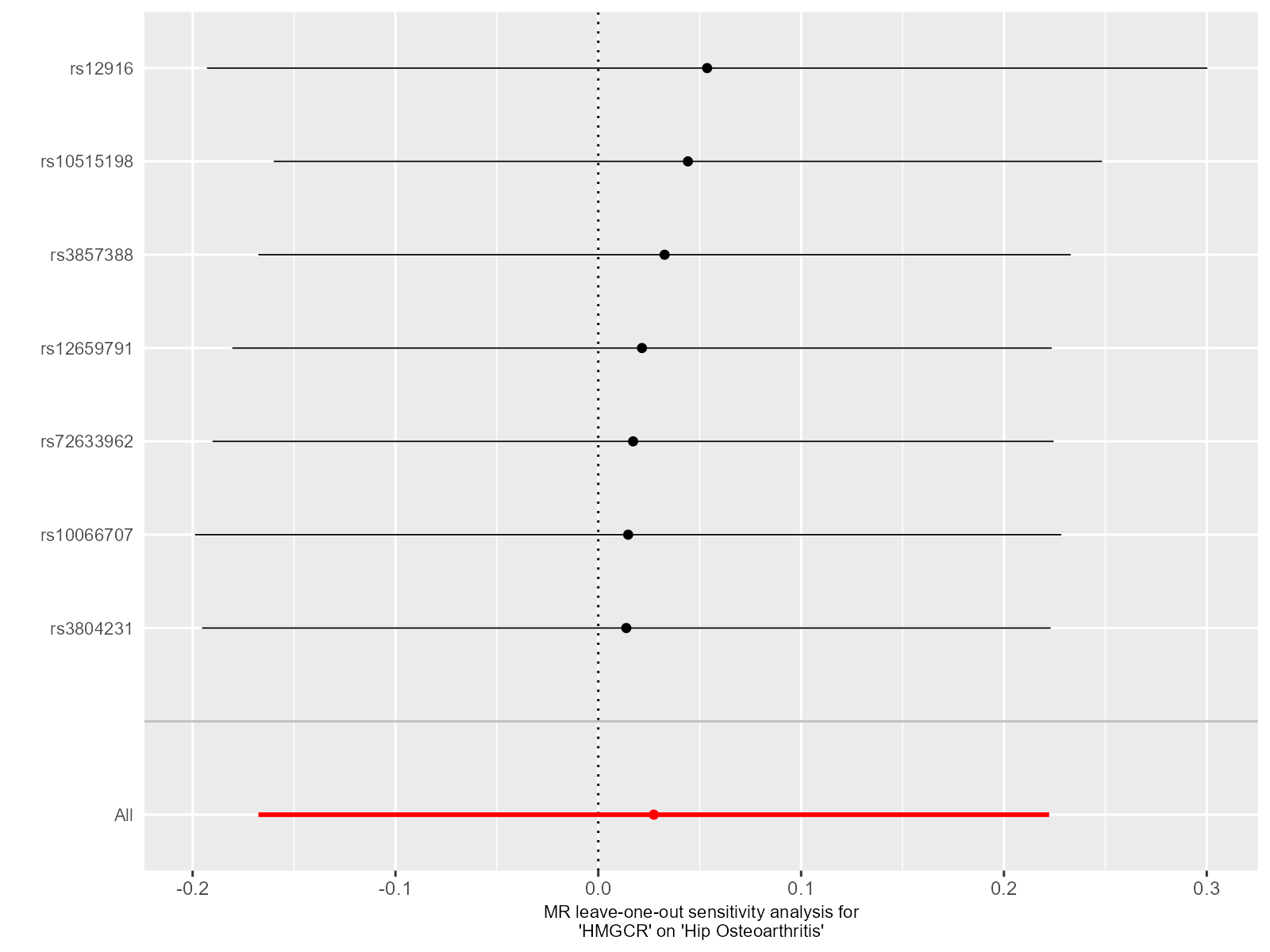
Supplementary Figure 12. Leave-one-out plot for sensitivity analysis of single SNP effect on** “**HMGCR”-to-“hip osteoarthritis” UVMR results.**

Leave-one-out plot using IVW method by sequentially re-evaluating the causal estimate after discarding one SNP at a time, which helps determine whether the overall effect is driven by the specific genetic variant. The black point denotes the causal effect estimate after discarding a certain SNP, and the black line signifies the 95% CI of estimate. The red point symbolizes the causal effect estimate, and the red line indicates the 95% CI of the estimate.

**
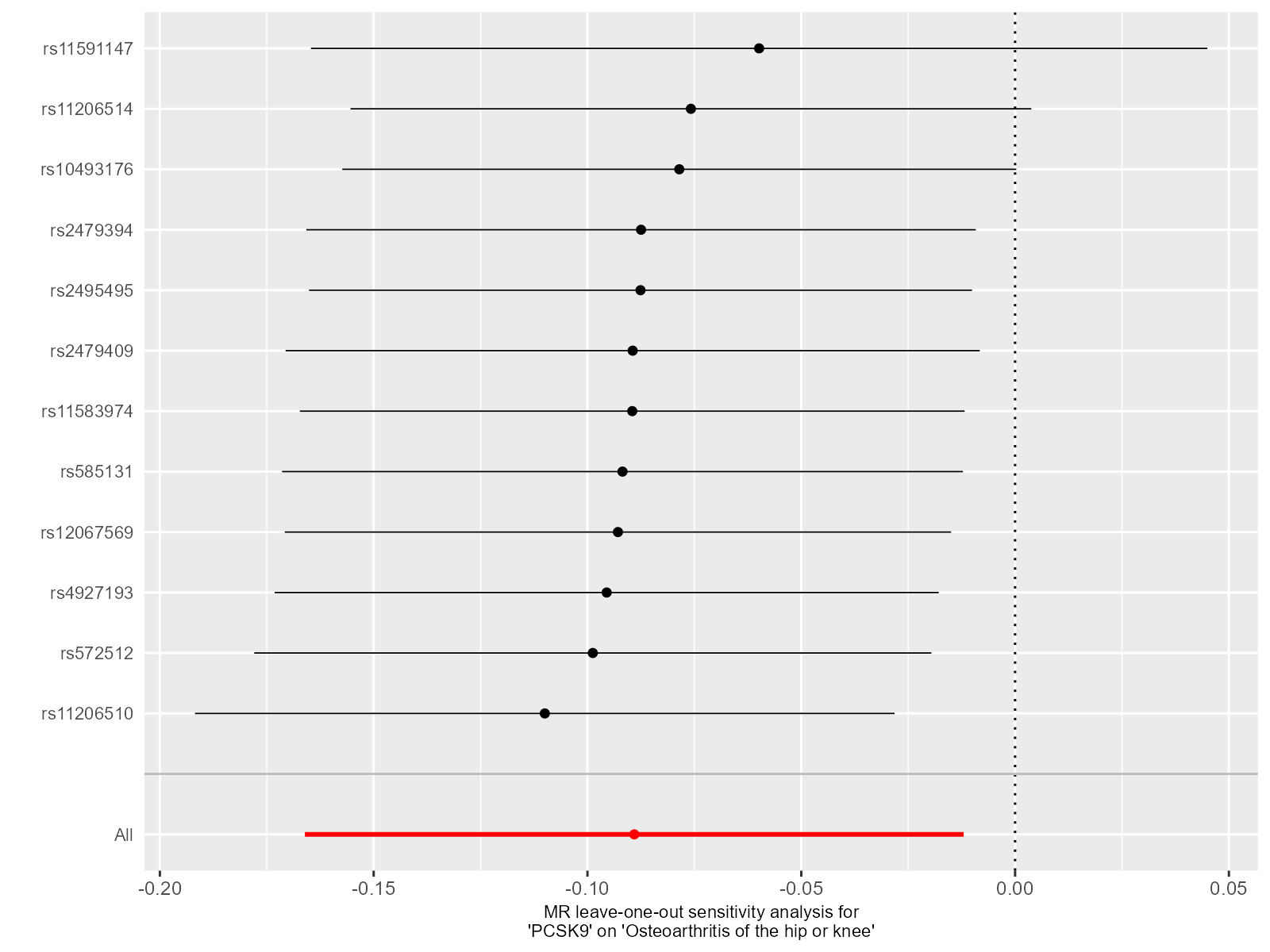
Supplementary Figure 13. Leave-one-out plot for sensitivity analysis of single SNP effect on** “**PCSK9”-to-“osteoarthritis of the hip or knee” UVMR results.**

Leave-one-out plot using IVW method by sequentially re-evaluating the causal estimate after discarding one SNP at a time, which helps determine whether the overall effect is driven by the specific genetic variant. The black point denotes the causal effect estimate after discarding a certain SNP, and the black line signifies the 95% CI of estimate. The red point symbolizes the causal effect estimate, and the red line indicates the 95% CI of the estimate.

**
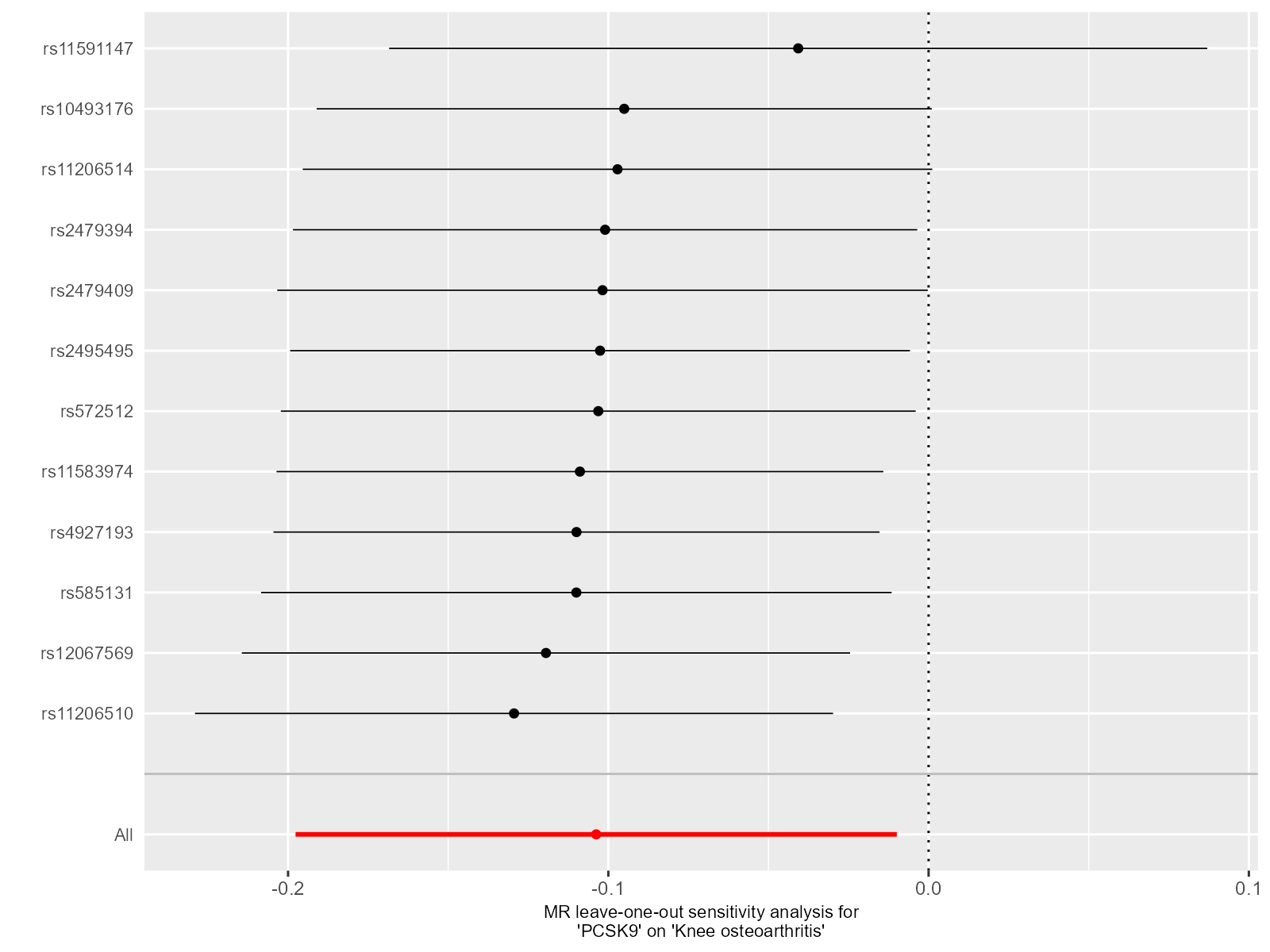
Supplementary Figure 14. Leave-one-out plot for sensitivity analysis of single SNP effect on** “**PCSK9”-to-“knee osteoarthritis” UVMR results.**

Leave-one-out plot using IVW method by sequentially re-evaluating the causal estimate after discarding one SNP at a time, which helps determine whether the overall effect is driven by the specific genetic variant. The black point denotes the causal effect estimate after discarding a certain SNP, and the black line signifies the 95% CI of estimate. The red point symbolizes the causal effect estimate, and the red line indicates the 95% CI of the estimate.

**
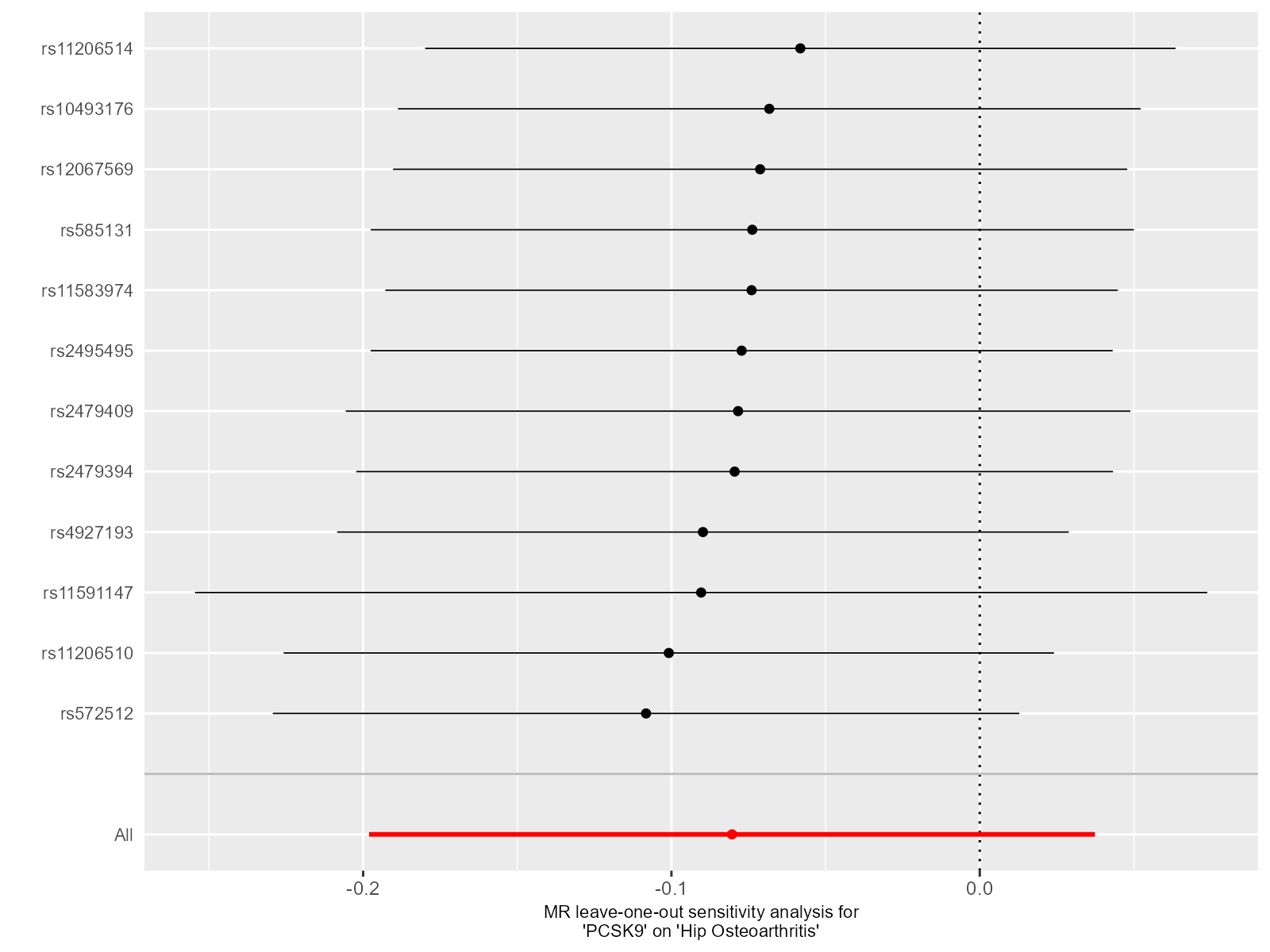
Supplementary Figure 15. Leave-one-out plot for sensitivity analysis of single SNP effect on** “**PCSK9”-to-“hip osteoarthritis” UVMR results.**

Leave-one-out plot using IVW method by sequentially re-evaluating the causal estimate after discarding one SNP at a time, which helps determine whether the overall effect is driven by the specific genetic variant. The black point denotes the causal effect estimate after discarding a certain SNP, and the black line signifies the 95% CI of estimate. The red point symbolizes the causal effect estimate, and the red line indicates the 95% CI of the estimate.

**
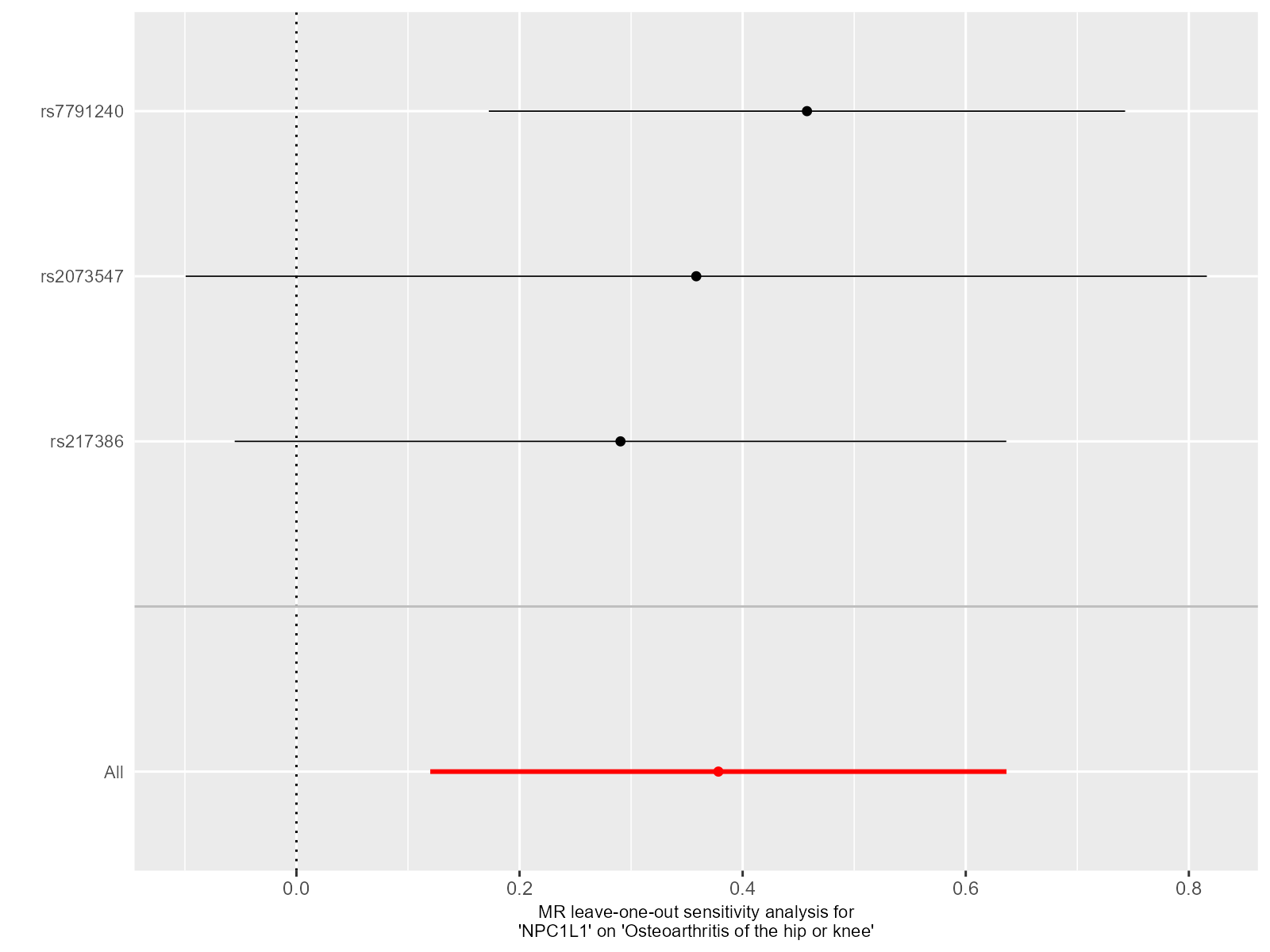
Supplementary Figure 16. Leave-one-out plot for sensitivity analysis of single SNP effect on** “**NPC1L1”-to-“osteoarthritis of the hip or knee” UVMR results.**

Leave-one-out plot using IVW method by sequentially re-evaluating the causal estimate after discarding one SNP at a time, which helps determine whether the overall effect is driven by the specific genetic variant. The black point denotes the causal effect estimate after discarding a certain SNP, and the black line signifies the 95% CI of estimate. The red point symbolizes the causal effect estimate, and the red line indicates the 95% CI of the estimate.

**
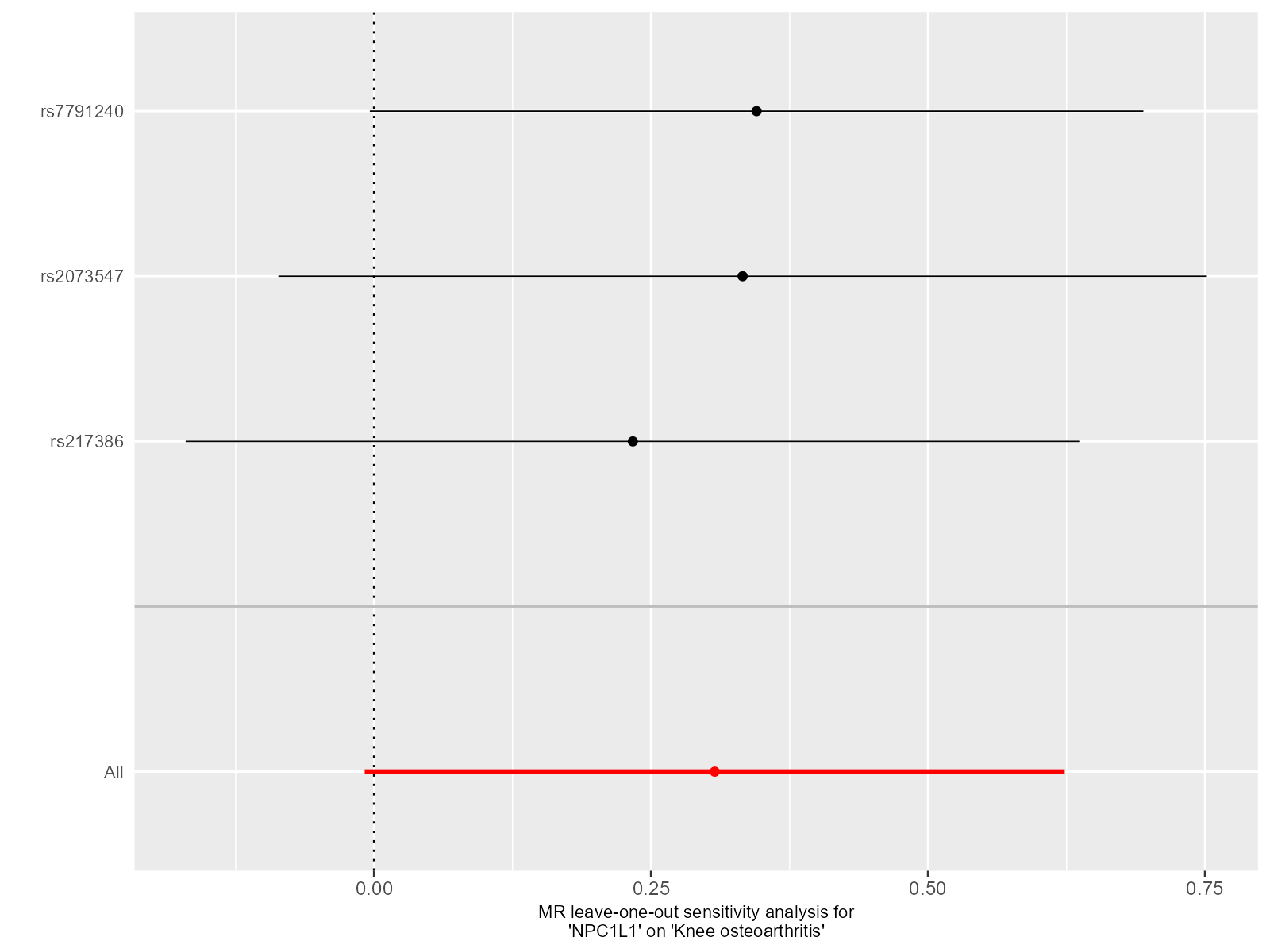
Supplementary Figure 17. Leave-one-out plot for sensitivity analysis of single SNP effect on** “**NPC1L1”-to-“knee osteoarthritis” UVMR results.**

Leave-one-out plot using IVW method by sequentially re-evaluating the causal estimate after discarding one SNP at a time, which helps determine whether the overall effect is driven by the specific genetic variant. The black point denotes the causal effect estimate after discarding a certain SNP, and the black line signifies the 95% CI of estimate. The red point symbolizes the causal effect estimate, and the red line indicates the 95% CI of the estimate.

**
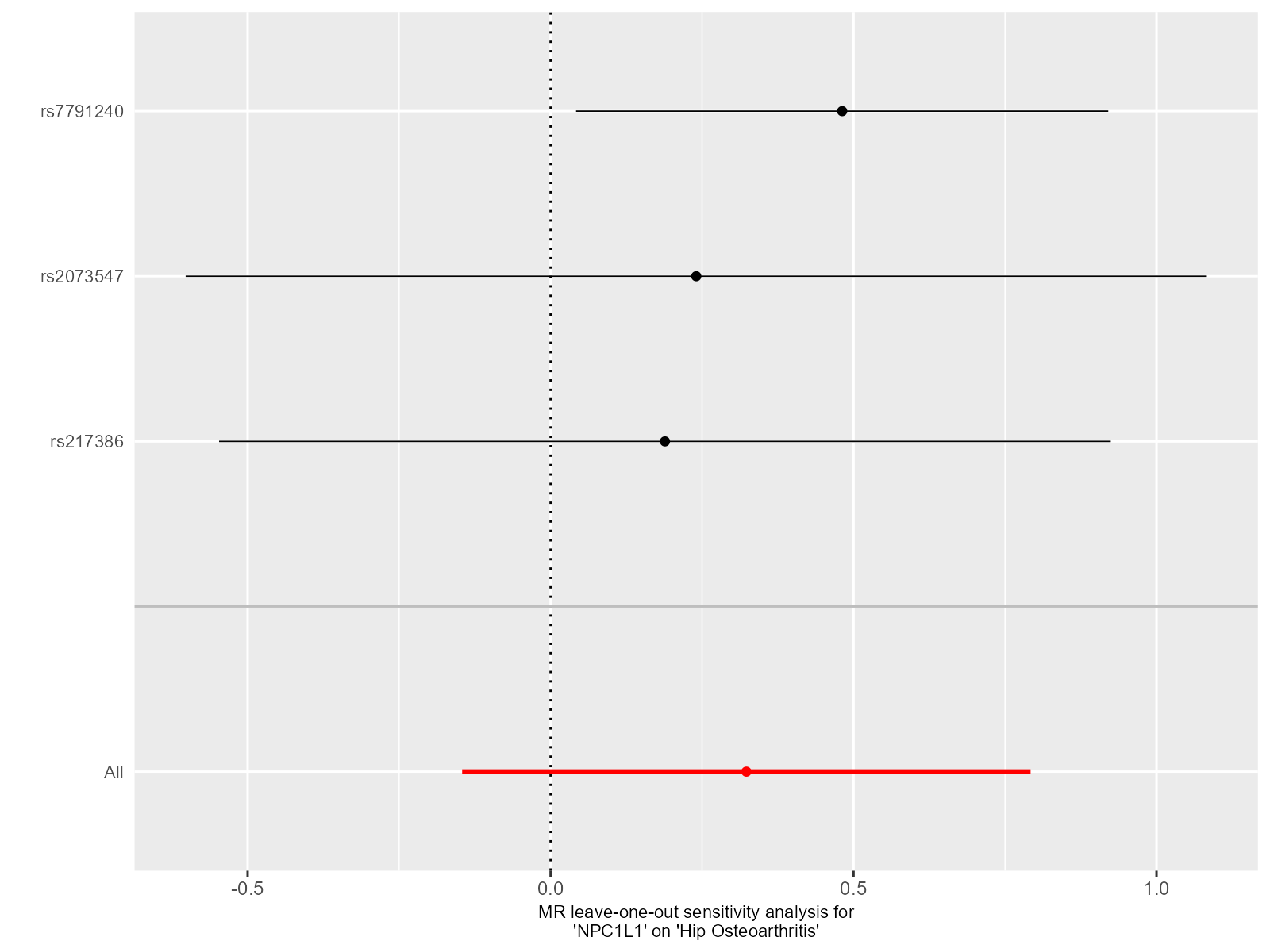
Supplementary Figure 18. Leave-one-out plot for sensitivity analysis of single SNP effect on** “**NPC1L1”-to-“hip osteoarthritis” UVMR results.**

Leave-one-out plot using IVW method by sequentially re-evaluating the causal estimate after discarding one SNP at a time, which helps determine whether the overall effect is driven by the specific genetic variant. The black point denotes the causal effect estimate after discarding a certain SNP, and the black line signifies the 95% CI of estimate. The red point symbolizes the causal effect estimate, and the red line indicates the 95% CI of the estimate.
